# Life-History Traits of the Miocene *Hipparion concudense* (Spain) Inferred from Bone Histological Structure

**DOI:** 10.1371/journal.pone.0103708

**Published:** 2014-08-06

**Authors:** Cayetana Martinez-Maza, Maria Teresa Alberdi, Manuel Nieto-Diaz, José Luis Prado

**Affiliations:** 1 Department of Paleobiology, Museo Nacional de Ciencias Naturales – CSIC, Madrid, Spain; 2 Molecular Neuroprotection Group, Hospital Nacional de Parapléjicos Toledo, Toledo, Castilla la Mancha, Spain; 3 UE INCUAPA, CONICET-UNICEN University of Olavarria, Olavarria, Argentina; Ohio State University, United States of America

## Abstract

Histological analyses of fossil bones have provided clues on the growth patterns and life history traits of several extinct vertebrates that would be unavailable for classical morphological studies. We analyzed the bone histology of *Hipparion* to infer features of its life history traits and growth pattern. Microscope analysis of thin sections of a large sample of humeri, femora, tibiae and metapodials of *Hipparion concudense* from the upper Miocene site of Los Valles de Fuentidueña (Segovia, Spain) has shown that the number of growth marks is similar among the different limb bones, suggesting that equivalent skeletochronological inferences for this *Hipparion* population might be achieved by means of any of the elements studied. Considering their abundance, we conducted a skeletechronological study based on the large sample of third metapodials from Los Valles de Fuentidueña together with another large sample from the Upper Miocene locality of Concud (Teruel, Spain). The data obtained enabled us to distinguish four age groups in both samples and to determine that *Hipparion concudense* tended to reach skeletal maturity during its third year of life. Integration of bone microstructure and skeletochronological data allowed us to identify ontogenetic changes in bone structure and growth rate and to distinguish three histologic ontogenetic stages corresponding to immature, subadult and adult individuals. Data on secondary osteon density revealed an increase in bone remodeling throughout the ontogenetic stages and a lesser degree thereof in the Concud population, which indicates different biomechanical stresses in the two populations, likely due to environmental differences. Several individuals showed atypical growth patterns in the Concud sample, which may also reflect environmental differences between the two localities. Finally, classification of the specimens’ age within groups enabled us to characterize the age structure of both samples, which is typical of attritional assemblages.

## Introduction

Understanding how biological evolution has unfolded and how environment has shaped organisms, communities or biomes depends upon our capacity to extract information from the fossil record. Morphological analysis of fossil bones has constituted the principal source of information in vertebrate paleobiology. Other approaches, however, such as genomic or histological analyses of fossil bones, can provide complementary data unavailable for morphological studies. Bone is a dynamic tissue that undergoes changes in its histological microstructure in response to phylogenetic, developmental, enviromental and physiological factors [1], ([2] and references therein). Indeed, bone paleohistology has proven to be a rich source of information with regard to deciphering aspects of the growth, life history and physiology of extinct vertebrates. In mammals, paleohistological analyses have provided clues regarding the development, biomechanics, ecology and physiology of several extinct taxa [1]–[9]. Nonetheless, there are as yet few studies in mammals compared with other taxa such as dinosaurs or aves, and many groups remain unexplored.

Equidae is a classic family in evolutionary studies, exhibiting a complex evolutionary history and a rich fossil record spanning the early Eocene to the present. Hitherto, only two studies have addressed the bone histology of this family [3], [5] (see Table 1). The first, by Enlow and Brown (1957), analyzed diverse skeletal elements (see Table 1) from the Oligocene *Mesohippus*, the Miocene *Merychippus* and *Parahippus blackburgi*, and the Plio-Pleistocene *Plesippus shoshonensis* (synonimous to *Equus simplicidens*). The second study by Sander and Andrässy (2006) [5] analyzed the tibia and the third metatarsal of the Late Pleistocene horses *Equus germanicus*, *Equus ferus* and *Equus hydruntinus*, all from Europe. Both studies provide descriptions of the bone tissue microstructure, but provide no further biological interpretations. According to these studies, bone histology in horses is characterized by plexiform bone tissue with variations in the vascular pattern and the presence of bone remodeling, mainly in the inner two-thirds of the cortex. Additionally, Sander and Andrssy (2006) [5] detected the presence of up to three lines of arrested growth or LAGs in *Equus*. These authors suggested that these growth marks could be used for individual aging (skeletochronology [10]), as well as for inferring physiological changes under variable climatic conditions. Additional information can be obtained from histological studies of modern horses (*Equus caballus*) [11]–[13]. These studies mainly focus on the mechanical properties of the limb bones –particularly metapodials– in order to explore the capacity of the skeleton to adapt to physical activity in racehorses. In addition, Stover *et al*. (1992) [11] analyzed in detail the histology of the third metacarpal in horses up to 8 years old, describing changes in bone microstructure and remodeling during the ontogeny, as well as showing the occurrence of external fundamental system (EFS) in individuals over two years old (Table 1).

**Table 1:** Summary of extinct and extant equids analyzed in previous studies.

Reference	Genus	Age	Bone	Bone tissue type	Growth marks
[3]	*Mesohippus*	Oligocene	Mandible	Reticular bone	not indicated
	*Merychippus*	Miocene	Mandible	Reticular bone	not indicated
	*Parahippus*	Miocene	Humerus	Primary plexifom	not indicated
			Rib	Dense harvesian	not indicated
			Phalanx	Primary reticular	not indicated
	*Plesippus*	Pliocene	Rib	Dense harvesian	not indicated
	*Equus*		Humerus	Dense harvesian	not indicated
			Rib	Dense harvesian	not indicated
			Metatarsal	Dense harvesian	not indicated
[5]	*Equus*	Pleistocene	Tibia	Plexiform bone	LAGs and EFS
			Metatarsal	Plexiform bone	LAGs and EFS
[11]	*Equus* 0–6 months		Metacarpal	Fibrolamelar bone	No LAGs/No EFS
	*Equus* 1–2 years		Metacarpal	Fibrolamelar bone	No LAGs/No EFS
	*Equus* adult 3–8 years		Metacarpal	Dense harvesian	No LAGs/EFS

The table shows genus, age, bone and data obtained from the histological analysis (bone tissue type and growth marks) in previous studies.

In order to gain knowledge on the life history, physiology, and growth patterns of equids during their evolutionary history, we analyzed the histology of the Neogene equid *Hipparion*. The genus *Hipparion* characterizes the Upper Miocene and Pliocene faunas of Eurasia. It represents an intermediate stage of horse evolution characterized by: i) a medium albeit highly variable body size; ii) a moderate-to-high degree of hypsodonty; and iii) a partial reduction of the autopodium to a major central toe and two reduced lateral ones (e.g [14]). In the present paper, we first describe and compare the histology (microstructure, vascular pattern, extent and distribution of bone remodeling, and number and distribution of growth marks) of a large sample of limb bones of *Hipparion concudense* from the upper Miocene site of Los Valles de Fuentidueña (Segovia province, Spain). These comparisons enabled us to establish the reliability of metapodials for skeletochronology. Based upon this assumption and on the abundance of third metapodials in fossil assemblages, we analyzed two Spanish Miocene populations of *Hipparion concudense* from different ages and environments [15]–[19], inferring the life history traits of this species and assessing the influence of different environments on its development. The histological data obtained allowed us to: i) identify different ontogenetic stages; ii) establish the growth pattern and the age of skeletal maturity of *Hipparion concudense*; and iii) propose tentative estimations of the individuals’ age at death and the population structure for both *H. concudense* samples.

## Materials and Methods

### Material

**Figure 1:**
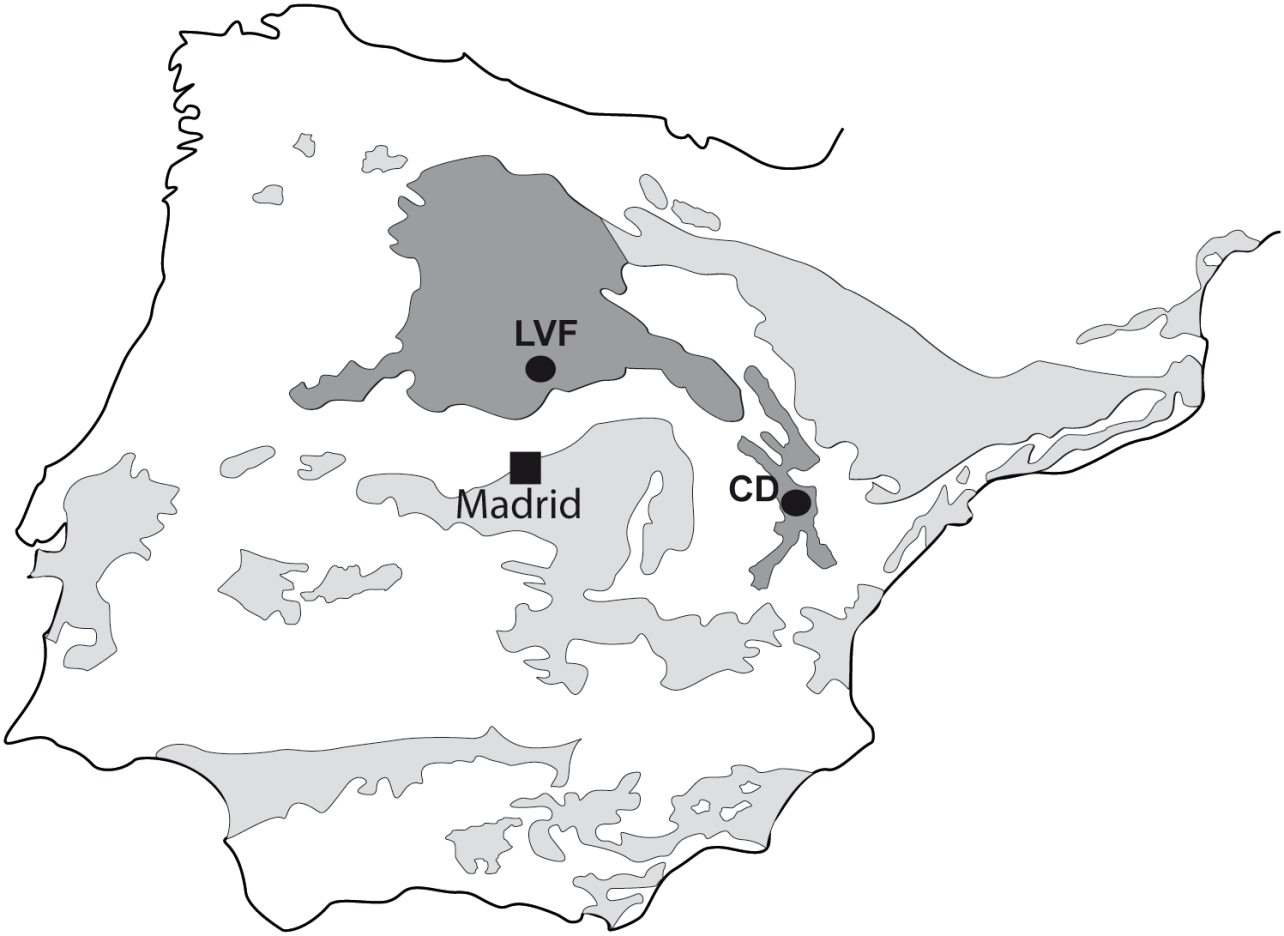
Geographic locations of the Miocene sites studied. The map shows the location of the two fossil sites studied in the present paper. LVF: Los Valles de Fuentidueña (Segovia, Duero Basin) and CD: Concud (Teruel, Calatayud-Teruel Basin).

In the preset study we analyzed a sample of limb bones from the upper Miocene localities of Los Valles de Fuentidueña (Segovia province, Spain) and Concud (Teruel province, Spain) (Figure 1) contained in the Vertebrate Paleontology Collection of the Museo Nacional de Ciencias Naturales– CSIC (Madrid, Spain). Los Valles de Fuentidueña (LVF) is an early Vallesian site in the Duero basin [15], whereas Concud (CD) is a Turolian locality in the northeast of the Calatayud-Teruel Basin [16]–[19].

To characterize the general histology of the limb bones, we used a sample of 3 humeri, 4 femora, 6 tibiae, 10 metatarsals, and 7 metacarpals (see Table 2 and Table 3) from the Los Valles de Fuentidueña site. All the material studied corresponds to unequivocally identifiable anatomical remains fragmented at the midshaft level to avoid damaging the most valuable and complete specimens. On the other hand, for the study of histological variability and skeletochronology, and the assesment of life history traits and growth patterns in two populations of *Hipparion concudense*, we focused on the central metapodials. Although previous studies in dinosaurs [20] have indicated that metapodials may be unreliable for skeletochronological studies, the comparisons performed in the present study show congruent results among the different limb bones (humeri, tibiae, femora and metapodials). We chose these anatomical elements because: i) they constitute the most common long bone in the fossil sites studied (and in most fossil sites) and ii) previous analyses of metapodial histology in extant horses, including the study of ontogenetic series, provide a good reference for comparison [11]. To conduct these analyses we added a sample of 9 metacarpals and 6 metatarsals from the Concud site to the LVF sample (Table 3). The samples from Valles de Fuentediueña and Concud include 21 specimens fragmented at the midshaft level and preserving the fully fused epiphyses (distal or proximal) and 11 anatomically recognizable diaphysis fragments lacking epiphyses (Table 3).

**Table 2:** Sample of the limb bones of *Hipparion concudense* from Los Valles de Fuentidueña.

Specimen	Element	LAG	EFS(max NGM)	Partial lines
30698	Femur	2	6	0
30699	Femur	2	7	1
30694	Femur	2	0	1
28594	Femur	2	4	0
30685	Tibia	2	3	0
30682	Tibia	2	0	0
30779b	Tibia	2	x	1
30684	Tibia	2	2	0
30686	Tibia	2	2	0
28826	Tibia	x	x	0
30779a	Humerus	2	4	0
30700	Humerus	3	2	0
30702	Humerus	2	3	1

The table shows data for the specimens analyzed for the comparative histological study. LAG: line of arrested growth; EFS: external fundamental system; max NGM: maximun number of growth marks within the EFS.

**Table 3:** The table shows data on each specimen of the two *Hipparion concudense* samples: bone section measurements and age at death.

Locality	Specimen	Region	LAG	EFS(max NGM)	Age atdeath	HOS	DensitySec. Ost.	APD(mm)	TD(mm)	CorticalArea(mm^2^)	MedullaryArea(mm^2^)	BoneArea(mm^2^)	CorticalThickness(mm)
**LVF**	30657 (355X)	MC	0	0	<1	1	2,49	21,072	25,990	373,164	68,726	441,891	8,617
	43971*	MT	0	0	<1	1	0.85	21,451	29,746	317,125	176,191	493,480	5,86
	30602*	MC	1	0	>1	2	4,1	21,601	24,885	348,103	83,249	431,352	9,238
	30613 (878)	MT	1	0	>1	2	6,48	23,508	26,109	379,688	103,555	483,243	9,701
	30613 (609)	MT	2	0	>2	2	8,998	25,061	28,085	435,307	120,043	555,351	7,531
	30656d (A311)*	MT	2	1 (2)	>4	3	15,967	26,149	25,807	449,423	107,047	556,470	7,818
	30656a*	MT	2	1 (2)	>4	3	3,9449	25,643	22,637	386,956	83,095	470,052	11,158
	30656c*	MC	2	1 (3)	>5	3	18,4	27,475	23,812	429,932	94,197	524,129	10,59
	30613-xfisura	MT	2	1 (3)	>5	3	2,39	24,561	24,699	408,082	82,646	490,728	8,661
	30613 (LVF6X)	MT	2	1 (4)	>6	3	14,519	29,157	26,130	465,484	127,547	598,548	9,878
	67917	MT	3	1 (5)	>7	3	5,87	26,640	26,031	420,688	120,533	541,221	7,444
	30599	MC	1	2 (3+3)	>8	3	4,3	19,908	23,048	279,782	86,823	366,605	7,366
	30613–599x	MT	1	1 (2)	>3 (>4?)	3	18,23	27,208	23,212	448,214	80,773	525,653	11,64
	30604**	MC	x	x	x	x	x	22,099	26,449	357,834	100,795	458,629	8,551
	67930**	MT	x	x	x	1	x	26,902	23,181	443,049	79,921	522,970	11,468
**CD**	17092	MC	0	0	<1	1	4,768	21,366	25,843	336,816	94,499	431,315	8,14
	31186*	MC	0	0	<1	1	2,208	17,853	25,979	289,917	79,351	369,268	5,172
	17197	MC	1	0	>1	2	6,173	23,249	24,308	325,942	96,997	418,52	9,364
	17188	MC	2	0	>2	2	7,191	20,565	26,717	318,95	99,652	418,603	7,13
	17093*	MC	2	1 (2)	>1	3	0,24	21,203	28,291	365,655	109,93	475,585	7,294
	17215*	MC	2	1 (2)	>4	3	5,01	20,908	27,122	348,189	91,328	439,516	7,627
	17083*	MT	2	1 (3)	>5	3	11,94	25,87	25,747	447,518	77,089	524,607	10,254
	17216	MC	2	1 (6)	>8	3	4,53	21,714	27,471	380,644	93,894	465,256	8,513
	17085	MT	2	1 (2)	>4	3	19,56	28,282	25,791	480,108	93,81	573,918	10,617
	17242	MC	1	1 (3)	>4	3	7,028	20,633	24,379	309,745	96,716	406,46	8,094
	17082	MT	1	1 (2)	>3 (>4?)	3	17,179	25,534	28,084	452,266	108,017	560,282	9,322
	31183 A	MT	1	1 (3)	>4 (>5?)	3	24,33	28,115	27,042	521,179	98,101	619,28	11,664
	31183 B**	MT	2	1	>2	3	x	29,986	27,521	646,621	137,266	509,356	11,356

LVF: Los Valles de Fuentidueña; CD: Concud; MC: metacarpal; MT: metatarsal; Asterisks indicate the degree of preservation: (*) the cortical bone is altered but histological features can be observed; (**) the cortical bone is highly altered and histological features are not discernible; LAG: line of arrested growth; EFS: external fundamental system; max NGM: maximum number of growth marks within the EFS; age at death was inferred from the numbers of growth marks; HOS: histological ontogenetic stage; ADP: antero-posterior diameter; TD: transverse diameter; Density Sec. Ost.: density of secondary osteons (number of secondary osteons/mm^2^).

### Preparation of histological sections

Histological sections were prepared from 1.5 cm-thick samples of the midshaft region following standard procedures [21]–[24]. Bone samples were embedded in epoxy resin EpoFix (Struers), the cutting surface was ground and polished with a Buehler low-speed Isomet with SiC grinding papers (SiC-800, SiC-1200; Struers) and fixed to a glass-slide with epoxy resin. Subsequently, 200 µm-thick sections were cut using a Struers Discoplan TS diamond saw, and finally ground and polished to a final thickness of 100 µm with the use of different SiC grinding papers (SiC-800, SiC-1200; Struers). All necessary permits were obtained for the described study, which complied with all relevant regulations (Vertebrate Paleontology Collection Department, Museo Nacional de Ciencias Naturales – CSIC, Madrid, Spain). All histological sections employed in the present paper are deposited in the Vertebrate Paleontology Collection of the MNCN and are available to researchers. High resolution images of the analyzed sections can be obtained from the corresponding author of this article.

### Analysis of the histological sections

Analysis and high-resolution imaging of the thin sections were performed using an Olympus BX61 transmitted light microscope equipped with an Olympus DP71 digital camera (Servicio de Microscopia y Analisis de Imagen, Hospital Nacional de Parapléjicos, Toledo, Spain). The required images were merged and processed with Adobe Photoshop CS3 (Adobe Systems Inc). The basic size measurements of the sections, including transversal and antero-posterior diameters, and the area of the full section, the cortical bone, and the medular cavity were obtained by means of the measurement tools implemented in the image J vs 1.47 m software [25].

The histological description of the cortical bone microstructure follows the terminology established by Francillon-Vieillot *et al*. (1990) [26] and de Ricqlès *et al.* (1991) [27]. We employed secondary osteon density (number of density osteons/mm^2^) to estimate the degree of bone remodeling. We calculated the density of secondary osteons by counting their number in 0.146 mm^2^ counting frames distributed according to a systematic random sampling scheme based on stereological criteria [28].

To conduct the skeletochronological analysis of the sample from which to infer the life history traits of *Hipparion concudense*, we analyzed the number of growth marks within the primary cortical bone [4], [6], [8], [10], [29]–[31]. We distinguished two types of growth marks: i) lines of arrested growth (LAG) -structures resulting from a cessation of bone growth during osteogenesis followed by a sudden resumption of growth- and ii) external fundamental systems (EFS) -set of avascular lamellar bone and several lines close to the periosteal surface [6], [10], ([31] and references therein), [32]. To be considered as growth marks, lines should surround the entire section [31], although in somes cases, remodeling may obscure the lines in restricted areas of the bone section. We took particular care in the identification of the growth marks, due to the presence of vascular patterns and color changes in the bone section that could be confused with growth marks.

Assuming that growth marks represent annual cycles in mammals (e.g. [4]–[6], [8], [30], [31]), we inferred the age of each specimen from the numbers of growth marks. We estimated the age of skeletal maturity as the number of LAGs until EFS following Chinsamy-Turan (2005) [1] and Chinsamy and Valenzuela (2008) [32]. This age might also correspond to the onset of sexual maturity in mammals, as proposed by some authors [34]–[36], but this issue remains controversial and depends on the availability of growth curves of the taxa under study to confirm this inference [30], [29], [37], [38]. In addition, some authors have considered the total number of rest lines (the number of LAGs and lines within the EFS) to estimate age at death in extinct mammals [35], [36], although, to our knowledge, there is no evidence to support this. Moreover, variations in the number of lines within the EFS in the specimens provides further uncertainity to this estimation. We therefore consider both the onset of sexual maturity and age at death as tentative estimations to be confirmed by means of future experimental analyses, and we provide the raw values of the numbers of growth marks, including the maximum number of lines in the EFS, for each individual, for future re-evaluation of these data.

## Results

### Histological characterization of the limb bones of *Hipparion concudense* (Los Valles de Fuentidueña)

**Figure 2:**
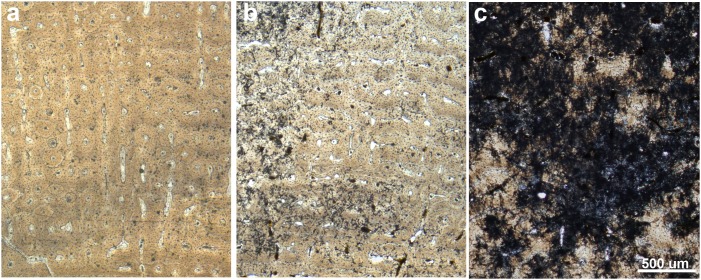
Different degrees of histological preservation in *Hipparion concudense*. The *Hipparion concudense* sample includes (a) specimens with a well-preserved histological structure 30656a (Mt), (b) specimens with taphonomical alterations that do not obscure the histological details 43971 (Mt), and (c) specimens that are badly damaged 30604 (Mc). The pictures show details of the cortical bone from the Los Valles de Fuentidueña specimens. Mt: metatarsal; Mc: metacarpal. Scale bar: 500 µm.

Bone tissue is generally well-preserved in the sample of *Hipparion concudense* from Los Valles de Fuentidueña. Nevertheless, two metacarpals (30602 and 30656c) and three metatarsals (43971, 30656d-A311, 30656a) present taphonomical alterations that do not obscure the histological details (Figure 2 and Table 3), whereas metacarpal 30604 and metatarsal 67930 are badly damaged, practically lacking any discernible histological feature (Figure 2c).

**Figure 3:**
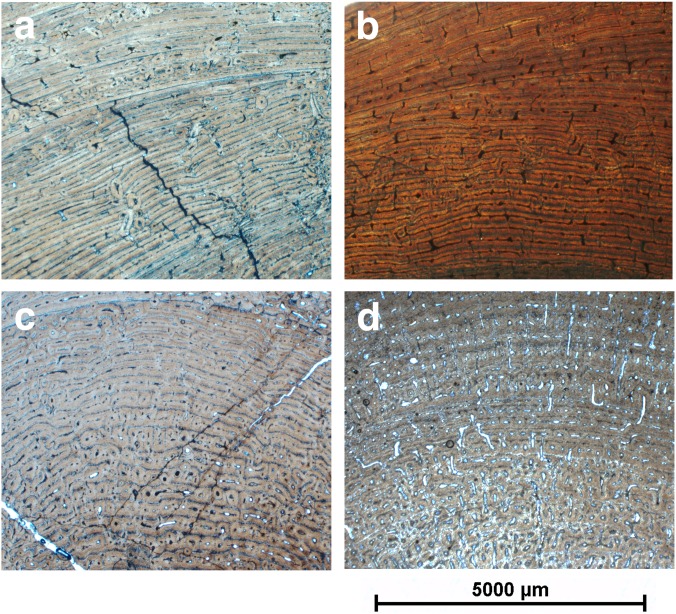
Bone histology of *Hipparion concudense* from Los Valles de Fuentidueña. Detail of the bone microsctructure observed in the transverse thin sections of mid-shaft from (a) humerus 30779a, (b) femur 30694, (c) tibia 30682, and (d) metapodial 30657-355x. Scale bar: 5000 µm.

Histological analysis reveals that all limb bones of the LVF sample are characterized by fibrolamellar bone tissue. However, the different limb elements differ markedly with regard to vascular patterns. Femora and humeri show a laminar bone tissue with vascular canals arranged circumferentially throughout the cortical bone (Figure 3 a and b). On the contrary, the tibiae are characterized by a laminar vascular pattern, except in the plantar region, where the bone shows longitudinal primary osteons arranged in circumferential rows, or is randomly distributed (Figure 3c). Finally, the metapodials are characterized by longitudinal primary osteons arranged in circumferential rows limited by thin layers of woven-fibered bone tissue (Figure 3d). In addition, some specimens show lamellar bone associated with growth marks in the periosteal (EFS) and the endosteal regions.

**Figure 4:**
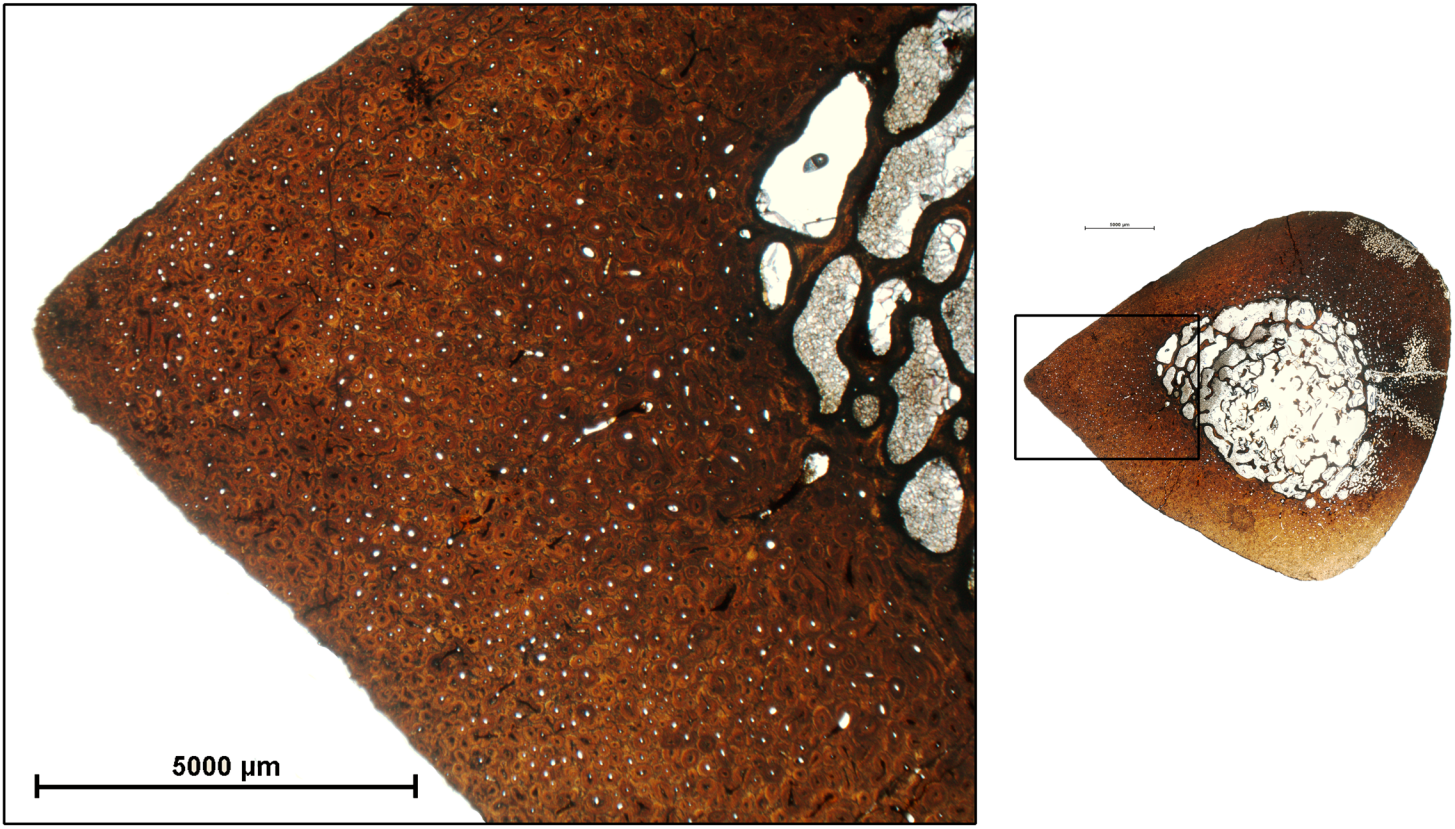
Detail of high bone remodeling. Tibia 28826 shows secondary osteons throughout the cortical bone that obscure histological features. Scalebar: 5000 µm.

**Figure 5:**
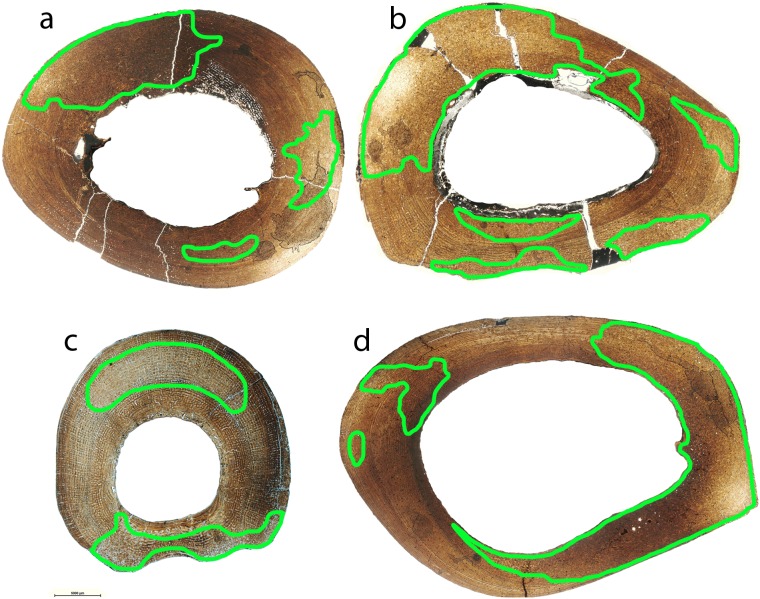
Distribution of bone remodeling areas in limb bones of *Hipparion concudense*. Green lines delimited the highly remodelled areas in each skeletal element. (a) humerus 30702, (b) tibia 38686, (c) metatarsal 67917, and (d) femur 30694. Scalebar: 5000 µm.

The primary cortical bone is highly remodelled in most of the LVF sample, particularly in tibia 28862 (Figure 4). Interestingly, the distribution of secondary osteons varies depending on the skeletal element. In the humeri, bone remodeling can be observed throughout the cortex of the dorsal region and in the middle cortex of the plantar region (Figure 5a). In the tibiae, secondary osteons are located in the outer cortex of the plantar region, throughout the cortical bone of the dorsal region, and in the outer cortex of the medial side of some specimens (Figure 5b). The femora present a high level of bone remodeling in the plantar region, from where it extends to other regions (Figure 5d). The large sample of metapodials reveals differences in the degree of bone remodeling, from unremodeled specimens to other highly remodelled ones, and specimens where remodeling is mostly restricted to the medial region of the cortex (Figure 5c). In both metacarpals and metatarsals, secondary osteons are mainly located in the dorsal region and in areas of the plantar region. Bone remodeling in the dorsal region is mainly restricted to the middle cortex, with few secondary osteons in the inner and outer cortex of some specimens, whereas in the plantar region, secondary osteons are located in the outer and the inner cortex of the regions coming into contact with the lateral metapodials.

**Figure 6:**
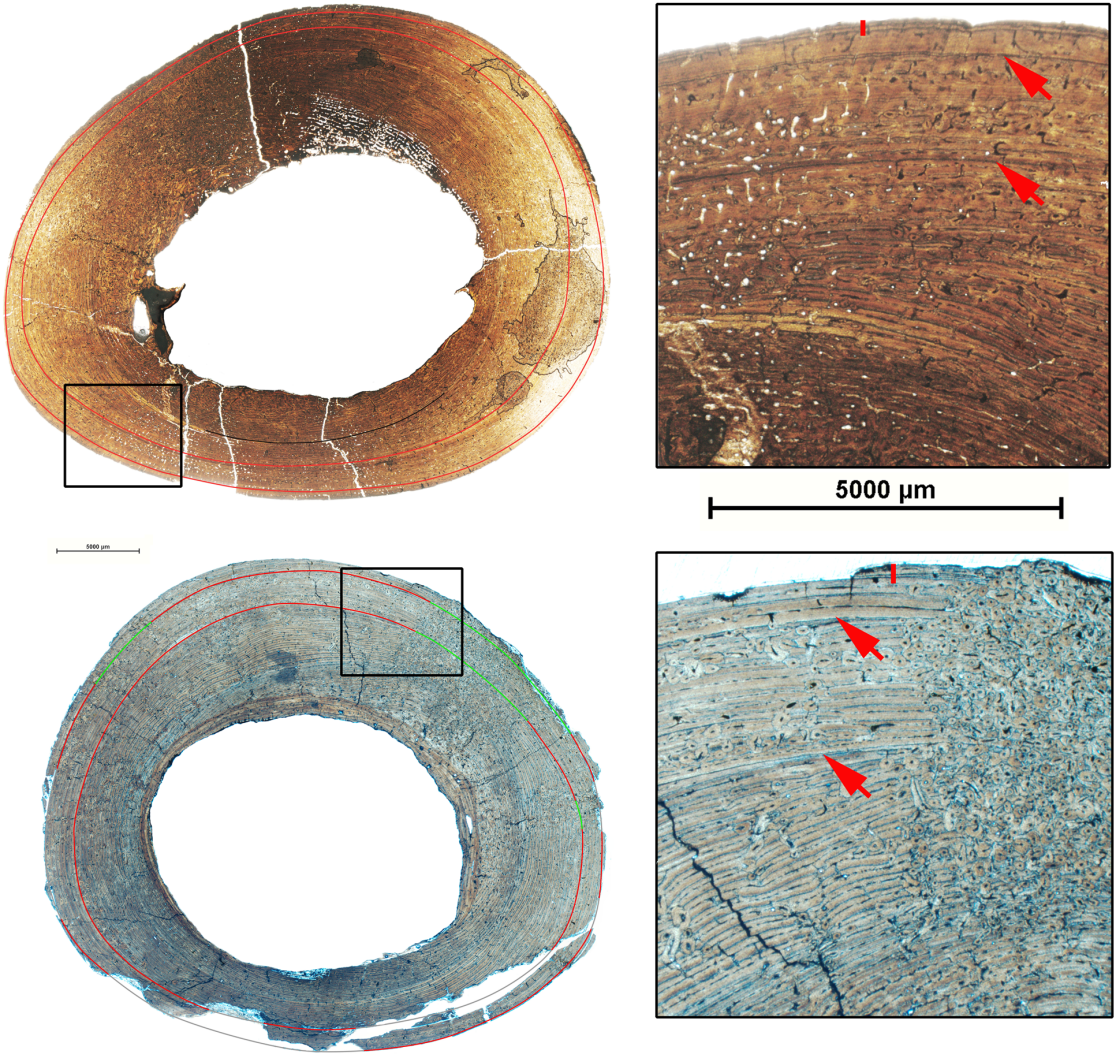
Growth marks in humeri of *Hipparion concudense*. The figure shows (on the left) the cross sections of humeri 30702 (top) and 30779a (down) with the lines of arrested growth identified in the cortical bone and (on the right) a detail of the cortical bone with these growth marks. Red lines are LAGs; green lines indicate the LAGs in the bone remodelled areas; gray lines show the inferred position of the LAGs in regions of the section lacking the cortical bone; black lines show the partial marks observed in the section; red arrows indicate the growth marks within the cortical bone; and vertical red lines show the EFS. Scalebar: 5000 µm.

**Figure 7:**
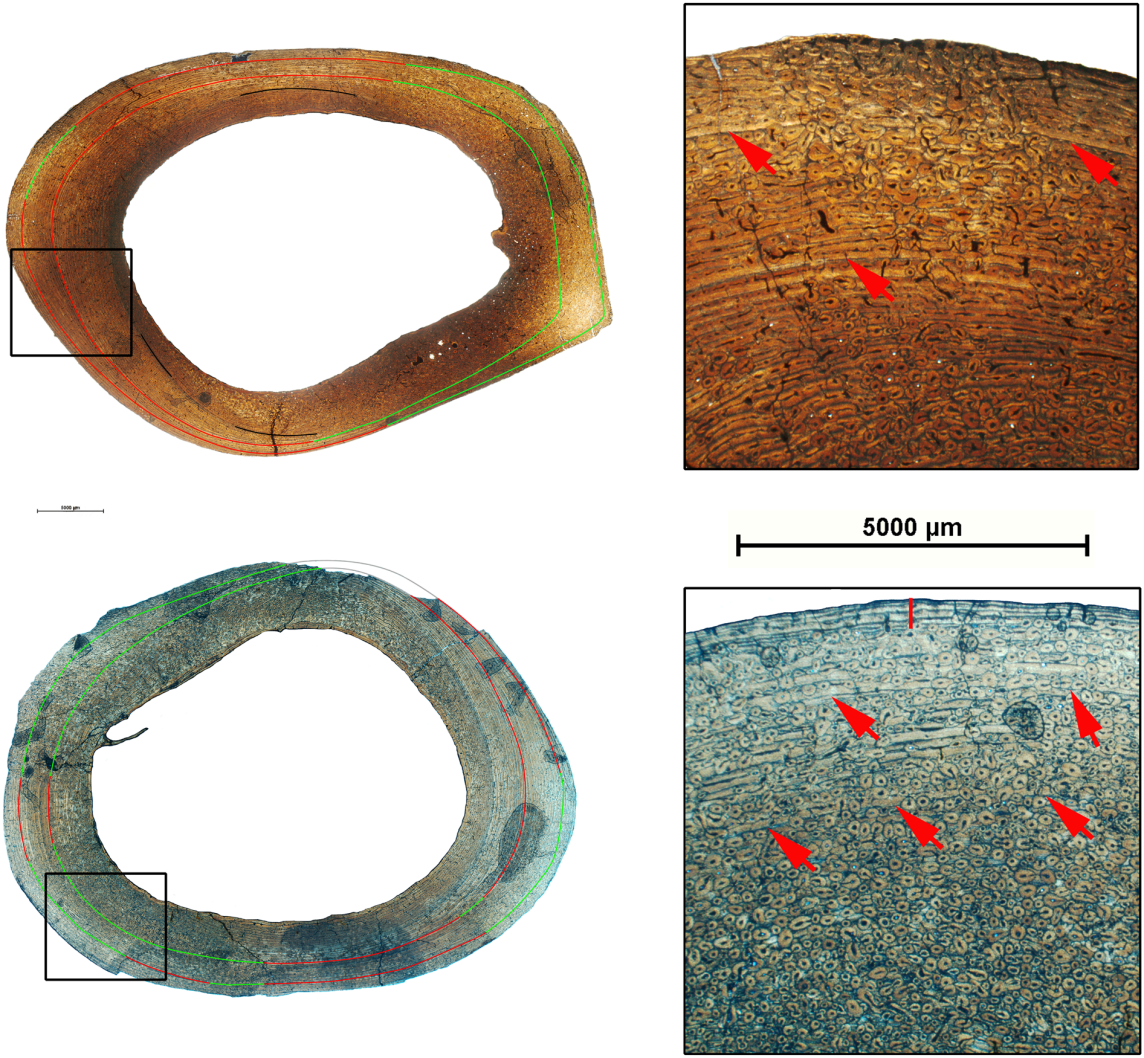
Growth marks in femora of *Hipparion concudense*. The figure shows (on the left) the cross sections of femora 30698 (top) and 30694 (down) with the lines of arrested growth identified in the cortical bone and (on the right) a detail of the cortical bone with these growth marks. Red lines are LAGs; green lines indicate the LAGs in the bone remodelled areas; grey lines show the inferred position of the LAGs in regions of the section lacking the cortical bone; black lines show the partial marks observed in the section; red arrows indicate the growth marks within the cortical bone; and vertical red lines show the EFS. Scalebar: 5000 µm.

**Figure 8:**
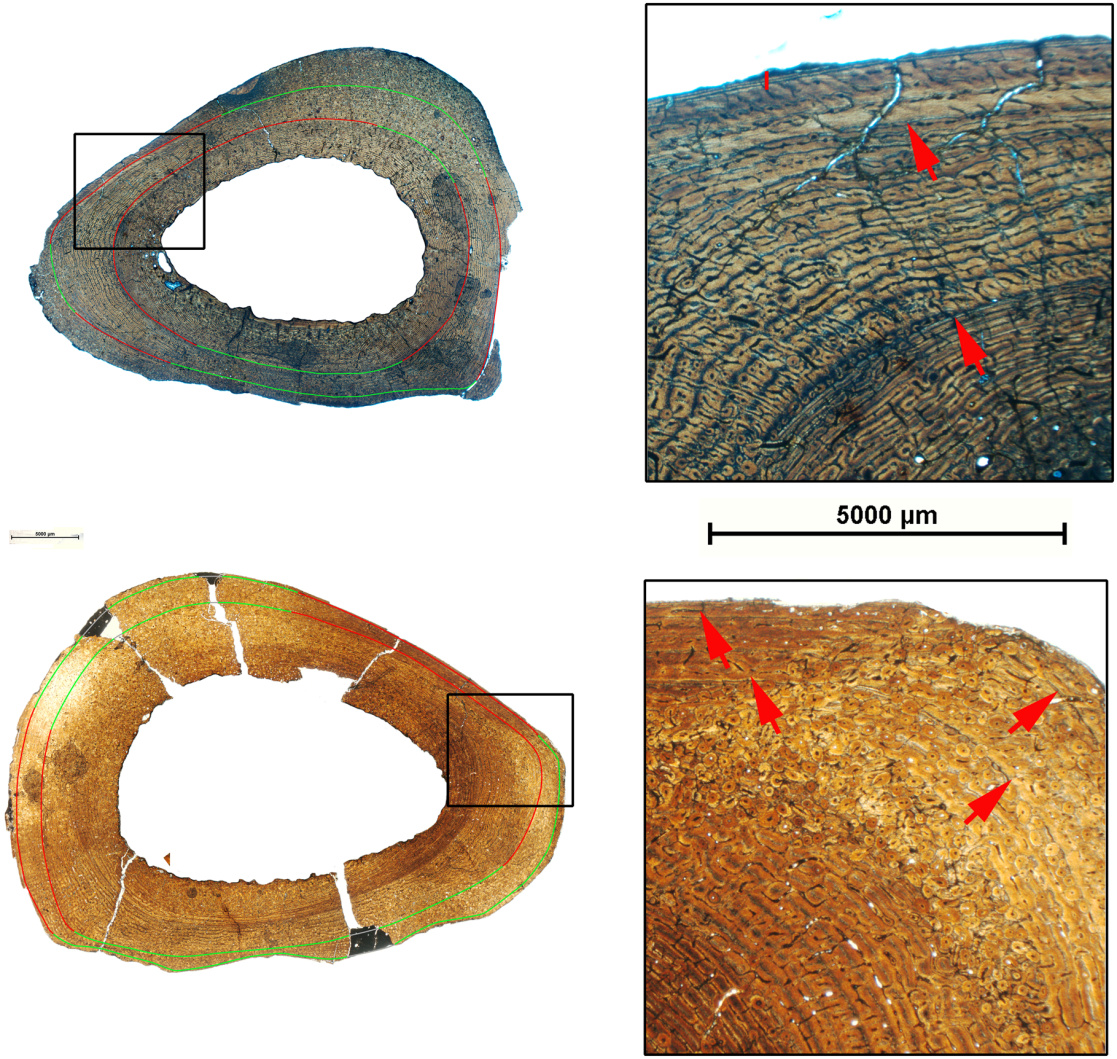
Growth marks in tibiae of *Hipparion concudense*. The figure shows (on the left) the cross sections of tibiae 30686 (top) and 30685 (down) with the lines of arrested growth identified in the cortical bone and (on the right) a detail of the cortical bone with these growth marks. Red lines are LAGs; green lines indicate the LAGs in the bone remodelled areas; gray lines show the inferred position of the LAGs in regions of the section lacking the cortical bone; red arrows indicate the growth marks within the cortical bone; and vertical red lines show the EFS. Scalebar: 5000 µm.

**Figure 9:**
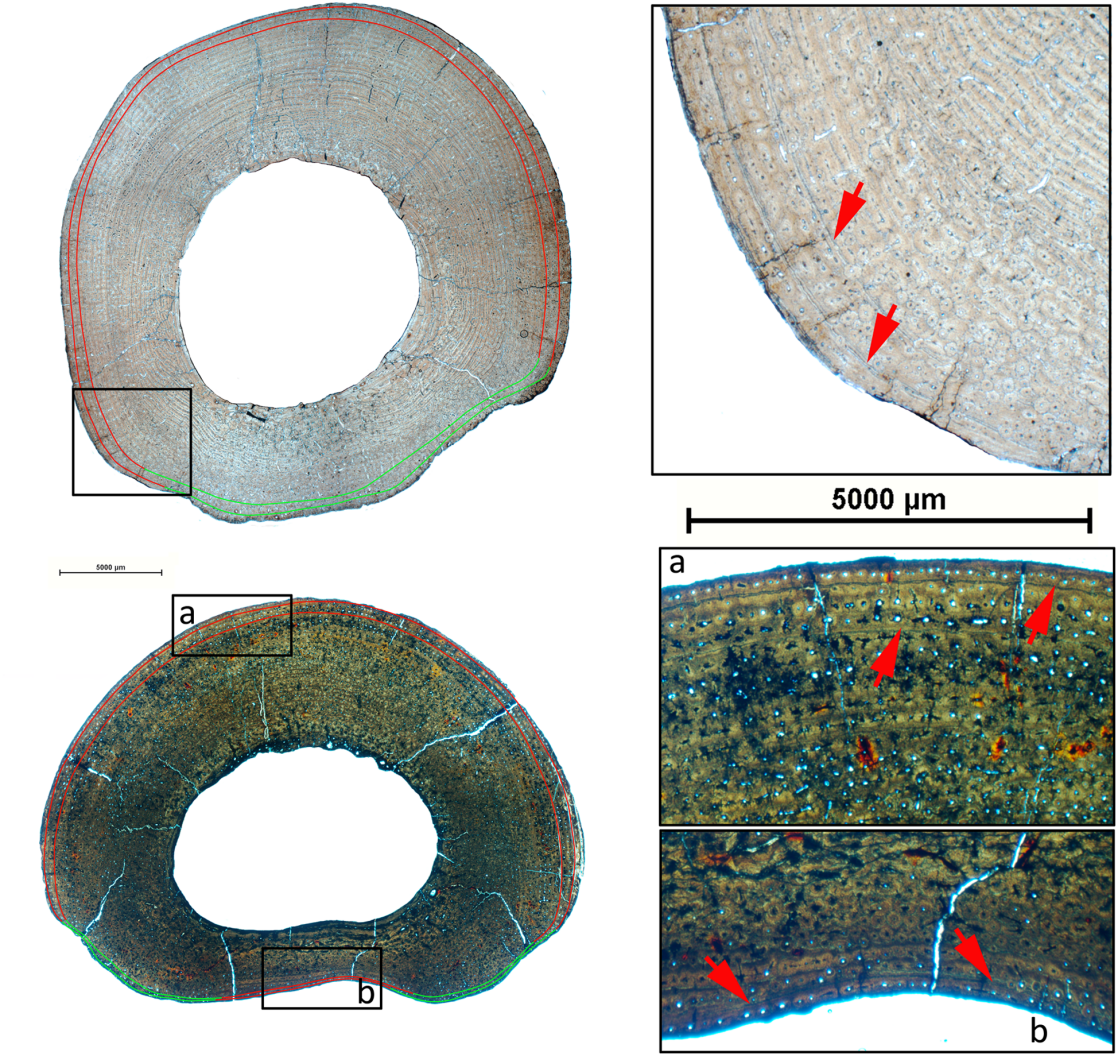
Growth marks in metapodials of *Hipparion concudense*. The figure shows (on the left) the cross sections of the metatarsal 30613-xfisura from Los Valles de Fuentidueña (top) and 17215 from Concud (down) with the lines of arrested growth identified in the cortical bone and (on right) a detail of the cortical bone with these growth marks. Red lines are LAGs; green lines indicate the LAGs in the bone remodelled areas; red arrows show the growth marks within the cortical bone. Scalebar: 5000 µm.

As illustrated in Figures 6–9, most of the specimens studied present two types of growth marks, lines of arrested growth (LAGs) and external fundamental systems (EFS). All the studied limb elements of *Hipparion concudense* exhibit the same general pattern in the distribution of growth marks, showing up to two LAGs with or without EFS (Table 2 and 3). The presence of EFS is associated with the presence of two LAGs, although several of the specimens reveal other patterns, such as metapodial 30613-599x, which presents one LAG plus one EFS, or metacarpal 30599, which presents one LAGs plus two EFSs (see Table 3). Moreover, humerus 30700 and metatarsal 67917 present three LAGs plus one EFS. In the humerus, LAGs are clearly distinguished across the whole section, whereas in the metatarsal, the second and especially the third LAGs can be distinguished from the EFS only on the lateral side. In some specimens, bone remodeling complicates the observation of growth marks in particular areas, although their continuity can be deduced after a detailed microscope analysis, except in the highly remodelled specimens 28826, 30604, 31183B and 67930. From a taphonomical point of view, it is worth mentioning that LAGs seem to be preferred regions for fractures within the cortical bone. In addition to LAGs and EFS, femora 30694, tibia 30779b and humerus 30702 also present partial lines within the primary cortical bones confined to a region of the section (Figure 6). In femur 30699 we observed a partial mark close to the medullary cavity which might be associated with cortical drift [31]. We did not consider them to be among the annual growth marks, but rather as non-cyclical growth marks such as cortical drift lines or temporary changes in the direction and relative intensity of bone deposition along a local section [31].

### Inferences of life history and developmental traits in *Hipparion concudense*: a comparative study of two populations

In order to infer the life history and developmental traits of *Hipparion concudense* and to identify temporal and environmental variations in its bone histology, we compared the histological features of the large sample of LVF metapodials with another large sample from Concud. Although the use of metapodials for skeletochronology is a controversial issue, our previous comparison of different limb bones demonstrates that they all provide coherent skeletochonological data.

**Figure 10:**
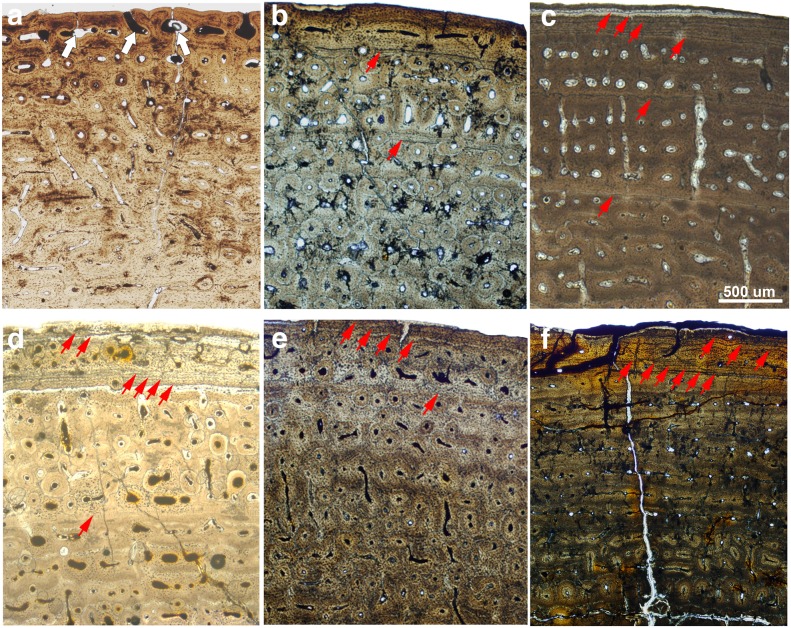
Bone histology of the metapodials of *Hipparion concudense* from Los Valles de Fuentidueña and Concud. The figure shows the histological features characterizing the three ontogenetical groups established in the *Hipparion concudense* sample from skeletechronological data (a–c) and different growth patterns (d–f). (a) 31186: immature specimens with vascular canals open to the periosteal surface (white arrows); (b) 17083: subadult specimen with fibrolamellar bone tissue, two LAGs and no periosteal canals; (c) 67917: adult specimen showing intracortical fibrolamellar bone with two LAGs and periosteal lamellar with EFS. Red arrows indicate growth marks; (d) 30599: one LAG, one inner EFS (4 LAGs) and one outer EFS (2 LAGs); (e) 17242: one LAG and one EFS (4 LAGs); 17184: (f) two EFSs (6 LAGs in the inner EFS and 3 LAGs in the outer EFS). Growth marks are indicated with red arrows. Scale bar: 500 µm.

As previously mentioned, the LVF sample of metapodials is histologically well preserved. In comparison, the bones from Concud (CD) are more fragile and difficult to prepare, but their histological features are nonetheless well preserved. As in LVF, some CD specimens presented slight taphonomical alterations which do not preclude histological analysis (see Table 3). In addition, specimen CD 31183B presents a high level of alteration but certain histological features could be identified (see Figure 2). The histological structure of the cortical bone in the Concud metapodials is similar to LVF, consisting of fibrolamellar bone tissue with circumferential rows of primary osteons limited by thin layers of woven-fibered bone tissue (Figure 10).

From the skeletochronological point of view, LVF and CD present two types of growth marks - lines of arrested growth (LAGs), as well as external fundamental systems (EFS). In the Concud sample, however, we observed that the LAGs are less marked than in the specimens from Los Valles de Fuentidueña; some CD specimens also exhibit annuli. In some of the CD specimens, LAGs are seen to surround the cross section (e.g. 17215, 17216, 17188) but in other specimens, these lines are weak in some regions of the section (e.g. 17083, 17093, 17242, 17085, 31183 B). In other specimens (31183A and 17082) the LAGs continue on the annuli, a narrow region of avascular bone reflecting a drastic decrease in bone formation rate, without growth being fully arrested [31] (Figure 10).

**Figure 11:**
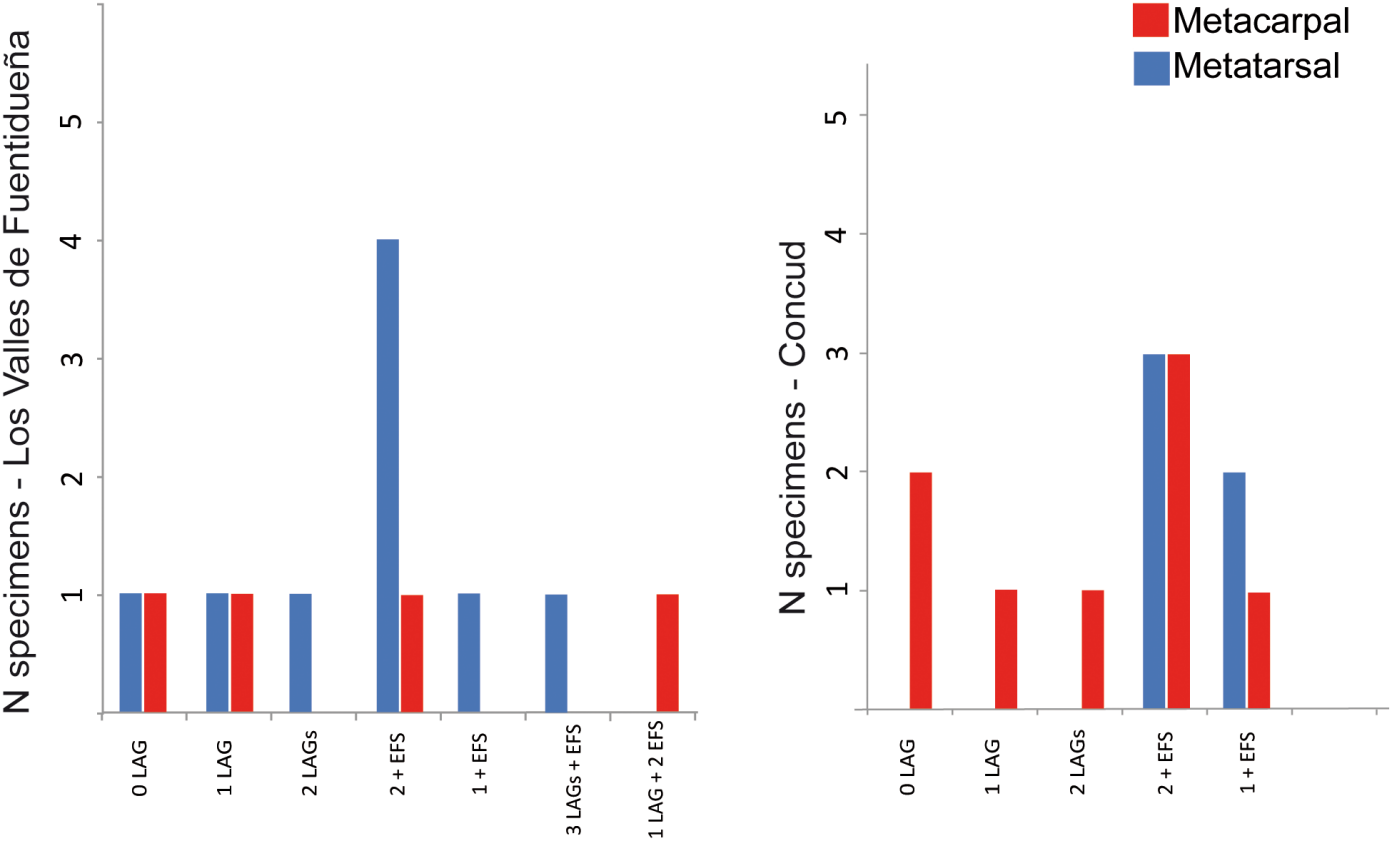
Distribution of metapodials in both populations of *Hipparion concudense*. The graph shows the number of metacarpals (Mc) and metatarsals (Mt) in the two samples from Los Valles de Fuentidueña (LVF) and Concud (CD), considering the number of growth marks. Red: metacarpals and Blue: metatarsals.

As illustrated in Table 3 and Figure 11, most specimens from both samples can be included in one the following: i) specimens lacking LAGs and EFS; ii) specimens with one or two LAGs; and iii) specimens presenting two LAGs plus EFS (Figure 10). Several specimens present atypical numbers of growth marks (Figure 10), including metapodials 17242, 17082 and 31183A from Concud and 30613-599x from Los Valles de Fuentidueña, which show only one LAG together with the EFS, and metacarpal 30599 from Los Valles de Fuentidueña, which presents one LAG plus two EFSs (see Table 3). In some specimens, the distance between LAGs and EFSs varies throughout the cross-section, and are so close to each other in some areas that the LAGs could be erroneously considered to be part of the EFS, whereas in other areas they are clearly separated (e.g. LVF:30656c and 30613 x-fisura; CD: 17197). As occurs in other limb bones (see above), LAGs (and sometimes EFSs) cannot be recognized in regions of some specimens due to a high degree of remodeling. Additionally, lines in the EFSs are sometimes lost due to erosion of the periosteal surface. Some specimens also exhibit rows of primary osteons limited by thin layers of different colours that could be misinterpreted as growth marks. These structures, however, are highly mineralized thin concentric plates of woven bone tissue that do not correspond to periodic growth marks [11].

**Figure 12:**
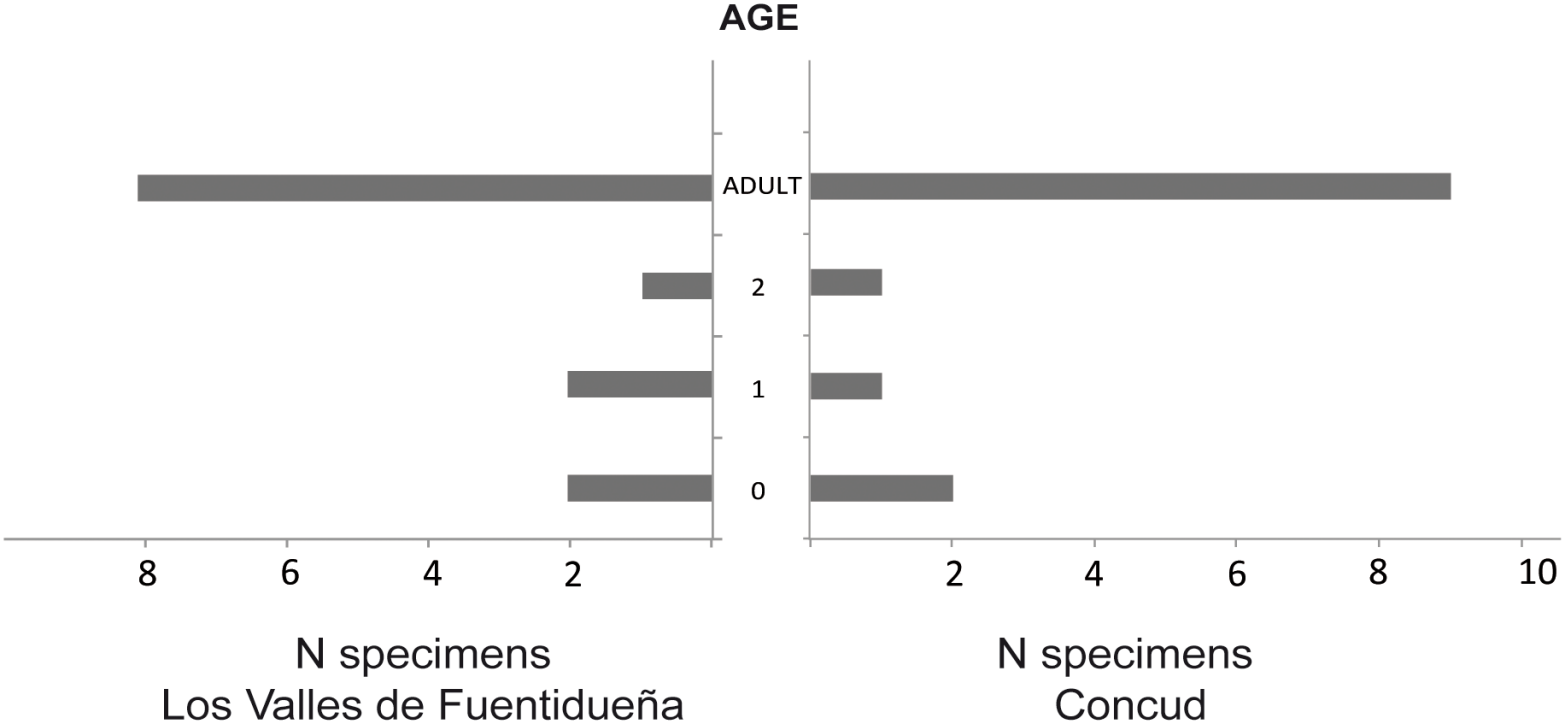
The age structure of the Los Valles de Fuentidueña and Concud samples. The high number of adults and the scarcity of two–year-old subadult individuals point to an attritional model, in which the specimens are acumulated in the assemblage due to a combination of factors mainly affecting the vulnerable age groups.

Considering LAGs as annual marks, we can distinguish specimens up to two years old (0, 1 and 2 LAGs) and specimens older than three years (2 LAGs plus EFS). As illustrated in Figure 12 (see also Table 3), the distribution of specimens among these age groups is similar in both localities, with most individuals in the adult group, whereas the least abundant group corresponds to 2-year-old individuals. Previous research has employed rest lines within the EFS to estimate age at death in mammal bones [35], [36]. According to these criteria, *Hipparion* lived as long as 8 years. However, as far as we know, there is no evidence to demostrate that rest lines within the EFS represent annual cycles. As a compromise, we determined the maximum number of rest lines observed in the EFS of each specimen and provided a tentative age estimate on that basis (Table 3), which should be validated in future studies addressing the biological significance of the EFS. The presence of two LAGs prior to the onset of the EFS in both the LVF and CD samples indicates that *Hipparion concudense* from these localities would generally grow for two years until reaching skeletal maturity at three years of age. According to some authors [34]–[36], EFS formation also marks the onset of sexual maturity or the age of first reproduction in mammals, but this inference is again controversial (see discussion). Some individuals, particularly in the Concud sample, present an alternative growth pattern reaching skeletal maturity in their second year of life (1 LAG before EFS; specimens LVF 30613-599x, CD 17082, CD 17242 and CD 31183A). Individual LVF 30599 shows an anomalous growth pattern with the presence of two EFS.

**Figure 13:**
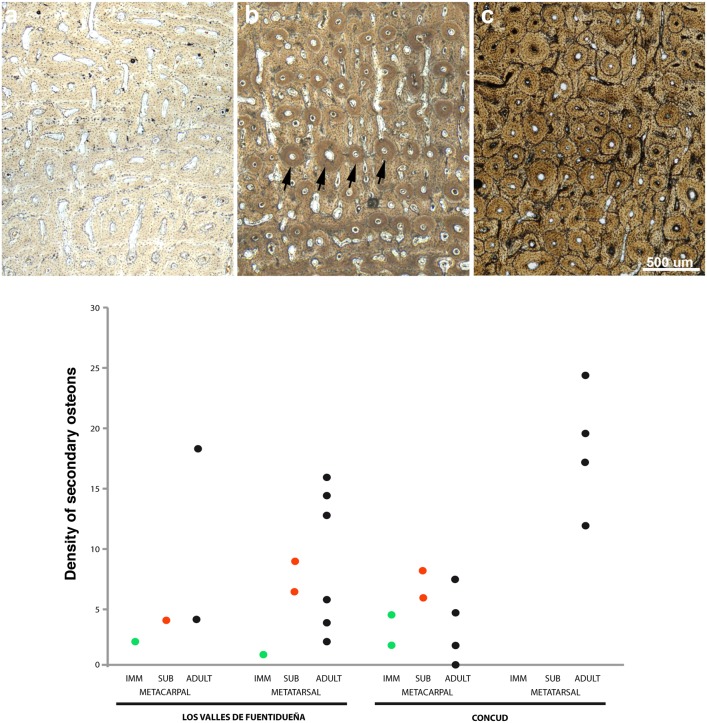
Different degrees of bone remodeling in *Hipparion concudense*. The figure shows different degrees of bone remodeling (a) specimen 30657-355x (Mc, LVF) shows a primary cortical bone with no secondary osteons; (b) specimen 67917 (Mt, LVF) shows an increase in bone remodeling; secondary osteons appear in the layer of woven bone tissue among rows of primary osteons (black arrows indicate a row of secondary osteons); (c) the cortical bone in specimen 30613-599x (Mt, LVF) is highly remodelled. Mt: metatarsal; Mc: metacarpal. Scale bar: 500 µm. (d) The graph shows the general increase in secondary osteon density in the ontogeny and differences between populations. The metatarsals from Concud are more remodelled than the metacarpals, whereas the metatarsals from LVF show a peculiar bimodal distribution.

Integration of bone histological structure and skeletochronological data reveals marked histological differences among the age groups identified. The first of these, comprising individuals lacking growth marks (less than one year old), is characterized by the presence of vascular canals open to the bone surface (Figure 10a) and slight or no bone remodeling with isolated secondary osteons (mean secondary osteon density: 2.579 secondary osteons/mm^2^; Figure 13a). Individuals from the second (one LAG, i.e. 1 year old) and the third (two LAGS, i.e. 2 years old) age groups are characterized by the absence of open vascular canals and lamellar bone in the periosteal surface (Figure 10b), except specimen 30602, which still presents open vascular canals, as in the previous age group. Bone remodeling patterns also change with respect to the first age group, from isolated secondary osteons to clusters of secondary osteons in the woven bone plates delimiting the concentric rows of primary osteons, mainly in the periosteal region (Figure 13b). Density of secondary osteons follows an increasing trend in the second and third age groups, with a mean of 5,584 secondary osteons/mm^2^ in specimens with one LAG and 8.09 in those with two LAGs. Finally, specimens older than 3 years are characterized by the presence of a layer of avascular lamellar bone in the periosteal surface corresponding to the EFS (Figure 10c) and the highest degree of bone remodeling (10.83 secondary osteons/mm^2^; Table 3 and Figure 13c), which sometimes obscures the presence of growth marks. The presence or absence of radial vascular canals, which has been related to a high bone growth rate [39], [41], appears to be randomly distributed among the studied specimens, irrespective of age, skeletal element, or locality.

While most histological features do not differ between localities or anatomical elements (metacarpals and metatarsals), detailed analysis of bone remodeling reveals several differences (Figure 13d). First, the Concud metacarpals show a lower level of remodeling than the metatarsals (Mt: 18,36 Sec. Ost./mm^2^, Mc: 3,36 Sec. Ost./mm^2^), and this remodeling only increases slightly during ontogeny. And second, the LVF metatarsals present a peculiar remodeling pattern in the older age group (2 LAGs plus EFS), exhibiting a bimodal distribution of specimens showing low and high degrees of remodeling.

**Figure 14:**
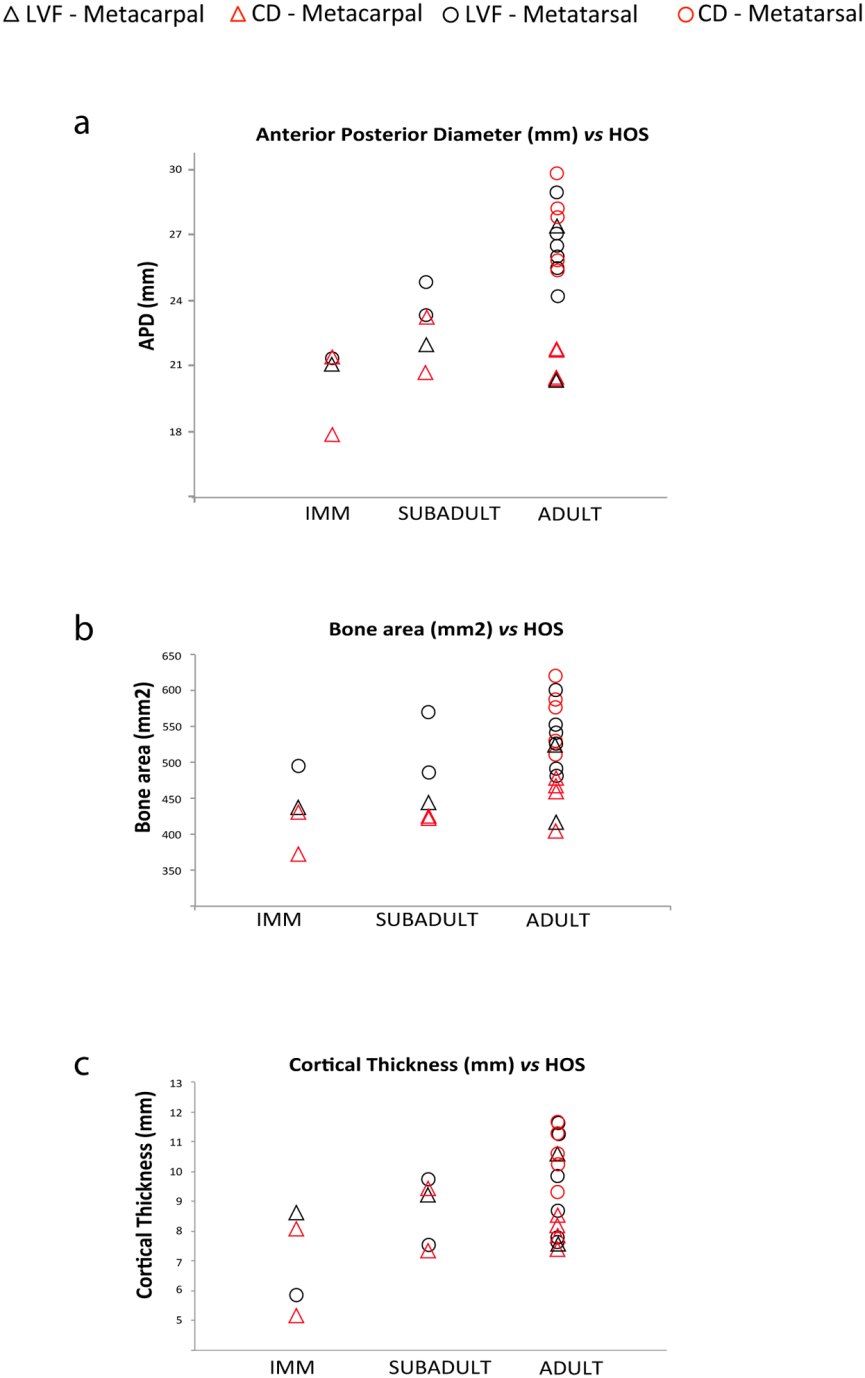
Bone section measurements and distribution of the specimens throughout the ontogenetic groups. The graphs show the increase in (a) antero-posterior diameter, (b) bone area, and (c) the cortical thickness throughout the three ontogenetic groups established in this study from skeletochronological data.

Ontogenetic trends in histological features are paralleled by size measurements of the bone sections –anterior posterior diameter (APD), cortical bone area and dorsal cortical thickness and, to a certain extent, bone section area–, which increase during ontogeny, although within-group variability is often greater than between groups (see Figure 14). On the contrary, the medullary cavity areas do not show marked differences among groups, whereas the transversal diameter appears to decrease throughout ontogeny.

## Discussion

In the present research, we conducted the first histological study of the limb bones of the Miocene equid *Hipparion concudense*. Our results reveal that all *Hipparion* limb bones show a similar histological structure, characterized by fibrolamellar bone tissue, but differ with respect to histological features such as vascular pattern or distribution of remodelled areas. Differences in vascular pattern involve the main orientation of the vascular canals, from the laminar bone tissue observed in humeri and femora, to the longitudinal vascular canals arranged in circumferential rows of the metapodials, and the laminar bone tissue with longitudinal canals in the plantar region of the tibiae. The different vascular patterns agree with previous observations in other equids, such as in the humerus of *Parahippus* [3], the tibiae and the metatarsals of Pleistocene *Equus* [5], and the humeri and the metapodials of extant *Equus* [3], [11]. The analysis of the vascular pattern in the long bones of the hadrosaurus *Hypacrosaurus stebingeri* by Horner et al. (1999) [20] (see also [23]) indicates that these differences can be related to the specific growth rate (Amprino’s Rule), size, growth dynamics or the biomechanical factors of each skeletal element.

Regarding bone remodeling, all the bones studied present secondary osteons, but their distribution in the cortical bone would appear to be specific to each limb bone. The remodeling patterns in these elements are similar to those described in extant and extinct *Equus* [5], [11] but differ from other perissodactyls (Rhinocerotidae) and mammalian taxa such as Proboscidea or Artiodactyla [5]. The ocurrence of bone remodeling in specific areas of the humerus, femur, tibia and metapodials in *Equus* and *Hipparion* is likely related to biomechanical factors shared by both genera. Considering the reduction of the number of toes characterizing equid evolution, it would be very interesting to analyze whether remodeling patterns are preserved in all equid species or whether they differ significantly in most plesiomorphic horses presenting completely developed lateral toes. Further studies addressing the biomechanics of limb bones will provide valuable information on the locomotion of *Hipparion* and the evolution of monodactylia in Equidae.

An interesting observation is the occurrence of growth marks –both LAGs and EFS– in the studied sample of limb bones of *Hipparion concudense*. The presence and distribution of growth marks differs from previous observations in extinct and extant horses. In a sample of extant horses analyzed by Stover *et al*. (1992) [11], growth marks resembling EFS appeared in the periosteal region of horses older than 29 months, but no LAGs were observed within the intracortical region. The absence of LAGs in these individuals indicates continuous growth during the first two and half years of life, which is likely due to the fact that the farm animals employed in the study were continuously fed and well cared for. On the other hand, in the Sanders and Andrassy (2006) [5] study of Pleistocene mammals, the authors observed up to 3 LAGs but no EFS in *Equus* tibiae and metatarsals. The presence of LAGs indicates arrested growth, whereas the lack of EFS suggests that either the sample studied by Sanders and Andrassy (2006) [5] only included subadult specimens, or that taphonomic or remodeling alterations in the periosteal region prevented identification of this structure. Growth marks reflect discontinuous growth, interrupted by periods of arrested growth that can result from internal endogenous factors or can be caused by environmental seasonality ([30] and references therein). The presence of growth marks in extinct wild equids [5] [present data] contrasts with the lack of LAGs in the farm horses studied by Stover *et al.* (1992) [11]. These differences suggest that environmental factors are most likely responsible for growth arrest and consequently, for the formation of growth marks. Indeed, several studies have shown that growth rates in extant horses are strongly influenced by several factors such as sex, birth month, weaning, feeding level, and the season of the year with growth patterns following changes in temperature and pasture growth [42]–[46]. For example, Martin-Rosset 2005 [46] shows that horses with a higher feeding level show a continuous growth curve, whereas those with lower feeding levels alternate periods of high growth with others of arrested or low growth. However, further research is needed to test whether changes in growth during ontogeny of extant horses caused by seasonal factors (e.g. temperature, food availability, photoperiod etc) and/or developmental events (such as weaning, puberty, sexual maturity or first reproduction) are reflected as growth marks within the cortical bone. Among these, photoperiod seems to play a key role in the regulation of bone growth rates [4], [11], [29], [45]–[47].

In addition to the histological characterization of *Hipparion* limb bones, our objective involved comparing different skeletal elements to evaluate the reliability of *Hipparion* metapodials for skeletochronology. Metapodials are generally the most abundant fossil remains of large mammals, including Perissodactyla, and they enable population level studies that are not possible with other, scarcer long bones. However, Horner et al. (1999) [20], (see also [23]) questioned the use of metapodials in skeletochronological studies, based on a detailed comparison of the number of growth marks in different skeletal elements of a single individual of *Hypacrosaurus*. The authors indicated that, among dinosaurs, metapodials present higher degrees of remodeling than other limb bones, which make them a poor source of data for skeletochronology [20], [23]. Contrary to these observations in dinosaurs, we observed a similar degree of remodeling in all limb bones, including metapodials. Across the LVF and CD samples, specimens present a variable degree of remodeling, from specimens presenting almost no secondary osteons to highly remodelled ones with most of the cortical bone covered by secondary osteons. With some exceptions, however, this remodeling variability does not reflect anatomical differences, but rather an ontogenetic gradient, in agreement with previous descriptions of high bone remodeling in adult specimens of extinct and extant *Equus* [3], [5], [11] and other extinct equids [3] but not in young horses (Stover et al., 1992) [11]. Horner et al. (1999) [20] also argued that the humerus, femur and tibia are the best skeletal elements for skeletochronology because they preserve the most complete record of growth marks. Conversely, we observed that the distribution of growth marks is similar among the different skeletal elements, including the metapodials. Even anomalous patterns –such as the presence of three LAGs together with one EFS- are observed in both the metapodials and the other skeletal elements. Furthermore, the metapodials show a higher degree of variability and anomalous patterns in growth marks than the other elements due to their much larger sample size. In summary, our results indicate that the different limb bones studied herein present comparable degrees of bone remodeling and, as far as we can establish in the present study, yield equivalent skeletochronological data. We therefore suggest that metapodials from *Hipparion* can be reliably used for skeletochronological analysis. Discrepancies with Horner *et al* (1999) [20] could be explained by the restricted growth of equids with respect to dinosaurs, which likely minimizes the effects of the differences in growth rates among skeletal elements. Moreover, we hypothesize that the presence of the same pattern of growth marks among long bones (including metapodials) of *Hipparion concudense* might also be influenced by the skeletal structure of their limbs.

Assuming that *Hipparion* metapodials constitute reliable elements for skeletochronology, we analyzed two large samples of central metapodials from the Miocene localities of Los Valles de Fuentidueña and Concud. In general terms, these skeletochronological results are broadly similar in both localities, characterized by up to two intracortical LAGs prior to the formation of the EFS. LAGs are marks caused by annual growth arrests which are used to infer the age of the specimen, whereas the EFS is a structure resulting from a general decrease in bone growth rate associated with the attainment of skeletal maturity. According to some authors [34]–[37], EFS also marks the onset of sexual maturity due to a trade-off between growth and reproduction [48], [49]. However, according to Lee *et al*. (2013) [50] reproductive maturity preceedes skeletal maturity in most vertebrates, including humans. In racehorses, skeletal maturity, defined as the formation of EFS, is achieved at two years of age [11], slightly later than the onset of puberty, estimated at 15 months. Full testis size, however, is reached in male horses years later, whereas skeletal maturation can occur at 6 years of age [51]. Thus, controversy regarding the agreement between the onset of sexual and skeletal maturity is not merely a matter of data, but also of how both sexual and skeletal maturity are defined.

Based on LAG and EFS definitions, these growth marks enable four age groups to be differentiated: individuals less than one year old, individuals one or two years old, and individuals over three years old which have already reached skeletal -and potentially sexual- maturity. Greater accuracy in the age estimates of the latter group can be achieved by considering lines within the EFS as annual growth marks [35], [36]. However, as we indicated previously, there is no evidence to support this proposal (e.g. [31]) and further studies are needed to establish their biological significance and to validate their use for age estimation. However, we tentatively consider these lines to provide an hypothetical age of death in specimens with EFS, observing individuals up to 10 years of age.

Skeletochrological aging of the LVF and CD individuals also enables us to interpret the histological variability observed in both samples from an ontogenetic perspective. Two features show consistent changes in central metapodials: the presence or absence of vascular canals open to the periosteal surface, and the presence or absence of lamellar bone in the periosteal region. Our results indicate that open vascular canals in the periosteal surface are usually observed in individuals without LAGs, i.e., in their first year of life, whereas older animals present closed vascular canals or even lamellar bone in the periosteal surface of the oldest individuals. In agreement, previous studies have shown that both features reflect ontogenetic changes in growth rate ([35] and cites there in). The presence of open vascular canals on the periosteal surface characterizes inmature horses [11] and other taxa [52]–[55]. According to Stover et al. (1992) [11], this type of bone, known as “saltatory primary osteonal bone” results from a rapid growth rate involving a particular mechanism described in detail by these authors. Stover et al. (1992) [11] added the term saltatory to stress its underlying growth mechanism that differentiates this type of bone from the otherwise similar “simple” primary osteonal bone. The latter type of bone develops later in young horses [11] and in *Hipparion concudense* specimens with one or two lags, but with no EFS. Finally, avascular lamellar bone resulting from a very slow growth rate [40], [50] is formed in the periosteal surface of older individuals that have reached skeletal maturity (i.e. presenting EFS), as occurs in extant adult horses over 29 months old [11]. We can therefore define 3 histological ontogenetic stages (HOS 1 to 3) in the *Hipparion* sample that broadly correspond to the inmature, subadult and adult horses described by Stover and colleagues (1992) [11]. Inmature individuals are characterized by fast-growing periosteal bone –“saltatory primary osteonal bone”-, whereas subadult individuals present a “simple” primary bone associated with a lower growth rate, which eventually almost ceases in the adult specimens when avascular lamellar bone is deposited in the periosteal region.

Bone remodeling also presents a high degree of ontogenetic variation in the *Hipparion* sample, showing a general increase in the density of secondary osteons with age. Inmature individuals present very low densities, which tend towards a progressive increase in subadults, reaching the highest values in adults. Within this general ontogenetic pattern, several differences between localities and between matacarpals and metatarsals become apparent. First, remodeling develops earlier in ontogeny in LVF than in CD; and second, the metacarpals from CD become less remodelled during ontogeny compared to the CD metatarsals and the LVF metapodials. These differences likely reflect biomechanical differences associated with environmental and/or morphological differences between the CD and LVF populations, such as those described by Pesquero and Alberdi (2012) [19]. According to these authors, LVF *Hipparion* are more slender than Concud individuals as a result of a drier environment and a harder substrate. Thus, it could be hypothesized that the hard ground of LVF might have induced a biomechanically stressful locomotion that caused high remodeling in both metacarpals and metatarsals. On the contrary, the soft ground of the wet environment described for CD might have caused less biomechanical stress, thus lowering bone remodeling, mainly in the metacarpal bone. However, there is a need for further studies analyzing the biomechanics of *Hipparion* in order to understand these differences.

All this histological and skeletochronological information, as well as the inferences derived therefrom, provide a relatively detailed reconstruction of the ontogeny, growth and life history of *Hipparion concudense*. This species had an initial, inmature stage of continuous rapid growth which extended during the first year of life. Subsequently, the individuals became subadults, a stage characterized by a reduced growth rate with cyclic periods of growth arrest. The subadult stage extended for 2 years prior to reaching maturity in the third year of life. This ontogenetic change was marked by a sharp decrease in growth rate marking the onset of skeletal, and possibly sexual, maturity [34]–[37]. Assuming that EFS marks the onset of sexual maturity and that rest lines within the EFS correspond to annual cycles, the resulting inferred life history of *Hipparion concudense* resembles that of extant equid species, but develops within a shorter time span. Sexual maturity is reached in or after the fourth year of life in extant equids (e.g. *E. burchelli*: 4 yrs and *Equus grevyi*: 6 years old [56]–[58] as well as in the Pleistocene *Equus* as indicated by the presence of subadult individuals over 3 years old (presenting 3 LAGS) in the sample analyzed by Sanders and Andrassy (2006) [5]. Lifespan is also prolongued in extant *Equus* species, reaching over 20 years in wild populations [58], [59]. Both disparities can be accounted for by size differences between the medium-sized *Hipparion concudense* (170–186 kg. [19]) and the larger *Equus* (e.g. *E. burchelli*: 220–322 kg (males), 175–250 kg (females), and *Equus grevyi*: 380–450 kg (males), 352–450 kg (females) [57]), in agreement with the well-established allometric relationships between body size and life history traits, including age of maturity and lifespan [56], [60].

In addition to this general ontogenetic pattern, we have observed alternative ones, particularly in CD. Indeed, half of the metacarpals of adult specimens from Concud, as well as one from LVF, present a single LAG in association with EFS. These individuals might have reached skeletal maturity in their second year of life, before the formation of the second LAG. Alternatively, it may indicate that these specimens arrested their growth only once prior to reaching skeletal maturity at the usual age (in the third year of life). The extent of this pattern among the Concud sample would therefore indicate that animals from this population encountered favourable environmental conditions that enabled more continous growth. At the other end of the spectrum, one LVF metapodial presents 3 LAGs together with EFS. This individual took one year more than the mayority of individuals to reach skeletal maturity, and reflects some intra-population variability in this life history trait. Specimen LVF-30599 shows a more anomalous pattern, characterized by the presence of two EFS. Considering that the presence of EFS corresponds to skeletal maturity [1], [33], the presence of two EFSs in this specimen is puzzling. Further research on life history and bone histology in wild extant horses could shed light on the significance of the EFS and the variability of bone growth and growth marks.

Age-at-death estimations obtained from the analysis of growth marks have enabled us to establish the age structure of the LVF and CD samples. The profiles from both localities are quite similar and do not appear to differ between metacarpals and metatarsals, although the sample is not large enough to reach a definitive conclusion. As illustrated in Figure 12, the profile of the age structure takes a U-shaped form, in which inmature (0–1 years) and adult individuals (above 3 years old) are numerous, whereas suabdults (2 years) are scarce. This profile corresponds to an attritional model, in which the specimens are accumulated in the assemblage due to a combination of factors (predation, neonatal mortality, disease, competition) that mainly affect the most vulnerable age groups [61]. In this respect, the profile of *Hipparion* from both the LVF and CD assemblages resembles the U-shape profiles of Pleiostecene *Equus* from archaeological sites in France [62], although the age classes are broader in *Equus*, likely due to its longer life span. Interestingly, the LVF and CD profiles differ from the profiles of Burchel’s zebras, but resembles those of the smaller bovid impala, both from current attritional accumulations sampled in the Akegara National Park (South Africa) [61].

In general terms, the present paleohistological analysis has yielded several interesting results, including the first description of the limb histology of the Miocene equid *Hipparion* and the inference of life history traits in two populations of this equid. An additional major finding involves the validation of the reliability of metapodials for skeletochronological analyses in this taxon. However, there is an urgent need for additional evidence in extant and extinct species to confirm the congruence of skeletochronological data among skeletal elements from the same individual and to determine to what extent this congruence occurs in other mammalian taxa. As we have mentioned throughout the text, further experimental data and other empirical evidence is urgently needed to verify many skeletochronological inferences employed in paleohistological studies. Nonetheless, our paper illustrates the richness of data that histological analysis can provide for the study of extinct populations. In this respect, histological features have enabled us to distinguish different age groups, characterize their growth pattern, estimate age of skeletal maturity, establish the variability in two populations and tentatively relate them to environmental differences in two samples of otherwise morphologically similar­ specimens. We therefore conclude that paleohistology is of great value for the study of extinct mammal populations and that this approach would strongly benefit from further experimental and empirical research with regard to understanding the biological basis of histological features.
